# Accelerated atrophy in dopaminergic targets and medial temporo-parietal regions precedes the onset of delusions in patients with Alzheimer’s disease

**DOI:** 10.1007/s00406-022-01417-5

**Published:** 2022-05-13

**Authors:** Riccardo Manca, Jose Manuel Valera-Bermejo, Annalena Venneri, for the Alzheimer’s Disease Neuroimaging Initiative

**Affiliations:** 1 Department of Life Sciences, Brunel University London, London, UK; 2 Department of Neuroscience, University of Sheffield, Sheffield, UK

**Keywords:** Alzheimer, Delusions, Dopamine, Nigro-striatal, MRI, Atrophy

## Abstract

People with Alzheimer’s disease (AD) and delusions have worse quality of life and prognosis. However, early markers of delusions have not been identified yet. The present study investigated whether there are any detectable differences in grey matter (GM) volume and cognitive changes in the year before symptom onset between patients with AD who did and did not develop delusions. Two matched samples of AD patients, 63 who did (PT-D) and 63 who did not develop delusions (PT-ND) over 1 year, were identified from the Alzheimer’s Disease Neuroimaging Initiative database. The Neuropsychiatric Inventory (NPI) was used to assess the presence of delusions. Sixty-three additional matched healthy controls (HC) were selected. Repeated-measures ANCOVA models were used to investigate group-by-time effects on the volume of selected GM regions of interest and on cognitive performance. No neurocognitive differences were observed between patient groups prior to symptom onset. Greater episodic memory decline and GM loss in bilateral caudate nuclei, medio-temporal and midline cingulo-parietal regions were found in the PT-D compared with the PT-ND group. A pattern of faster GM loss in brain areas typically affected by AD and in cortical and subcortical targets of dopaminergic pathways, paralleled by worsening of episodic memory and behavioural symptoms, may explain the emergence of delusions in patients with AD.

**Supplementary Information:**

The online version contains supplementary material available at 10.1007/s00406-022-01417-5.

## Introduction

People with Alzheimer’s disease (AD) often experience neuropsychiatric symptoms, especially in the more advanced stage of the disease [1, 2]. A subgroup of approximately 30% of patients may present with psychoses [3] (i.e. delusions and hallucinations). These symptoms represent a challenge to patients’ management since they recur in about 57% of cases [4] and are differentially associated with worse cognitive decline that is particularly severe in patients with hallucinations [5, 6]. Moreover, psychotic symptoms represent a risk factor for institutionalisation, particularly among patients with delusions [7], and are associated with higher caregiver burden [7–9]. Delusions, defined as false beliefs maintained despite contrary evidence, are the psychotic symptoms most commonly observed in people with AD with a prevalence almost twice that of hallucinations [3, 10]. Both misidentification and persecutory beliefs have been reported in this clinical population and they seem to represent two partially distinct clusters of psychotic symptoms, as stressed also by the most recent research and clinical criteria for psychosis in AD [11–13]. Moreover, psychoses appear to affect particularly psychosocial functioning [14], probably because patients with delusions have been found to present with a behavioural profile that is more extensively compromised than that of patients without delusions [5, 15].

The neurobiological substrates of psychotic symptoms in AD are still poorly understood. *Post-mortem* studies have highlighted that psychotic symptoms are frequently associated with comorbid non-AD neuropathology, in particular with the presence of Lewy bodies and cerebrovascular damage, rather than with AD-related pathology [16–18]. Palmqvist et al. [19] found that lacunar lesions to the left basal ganglia predicted increased odds of psychotic symptoms in patients with AD. However, patients presenting exclusively with AD neuropathology in Braak stages V/VI also had increased odds of delusions and these symptoms were associated only with neurofibrillary tangle burden and not with amyloid-β pathology [20].

Cross-sectional neuroimaging investigations of delusional AD patients have shown damage across multiple cortical and subcortical regions. Voxel-based morphometry analyses of structural magnetic resonance imaging (MRI) found lower grey matter (GM) volumes in the right fronto-parietal cortices and left claustrum [21], right hippocampal regions [22], left orbito-frontal and superior temporal cortices [23] in AD patients with delusions compared with those without. Moreover, Tetreault et al. [24] found that delusions in AD were associated with atrophy in bilateral ventrolateral frontal, orbitofrontal, and superior frontal cortices. Using an atrophy network mapping approach applied to resting-state functional MRI, these authors also found that delusions were associated with functional alterations in the same areas. Qian et al. [25], instead, observed that delusional patients with AD had reduced resting-state functional connectivity of the default mode network (DMN) in the left inferior parietal lobule. Single-photon emission computed tomography studies have found that patients with AD who experienced delusions had diffuse and predominantly right-lateralised hypoperfusion in frontal, temporal and parietal cortices [26–32], as well as in subcortical GM nuclei [33], with partially dissociable correlates for different subtypes of delusions. Similarly, investigations based on positron emission tomography (PET) showed metabolic alterations in consistent cortical and subcortical GM regions associated with delusions in AD [34–36]. Moreover, using a radiotracer selective for dopamine receptors, Reeves et al. [37] found upregulation of dopaminergic function in the striatum in patients with AD and delusions.

A few longitudinal studies have also been carried out to investigate neuroimaging parameters predictive of brain changes over a period of time associated with the emergence of AD-related delusions. Koppel et al. [38] found that orbitofrontal hypometabolism in patients with AD is not detectable prior to psychosis onset, but only after symptomatic manifestation. In contrast, several brain structural alterations were observed in patients who subsequently developed delusions over 1–2 years after MRI assessment; in detail: lower volume in medio-temporal, cingulate, insular and orbito-frontal GM [39]; lower fractional anisotropy in left parieto-occipital temporal and callosal white matter (WM) tracts [40]; and higher WM hyperintensity volume [41]. Using a simple within-group *t* test on a sample of 24 patients with AD and delusions from the Alzheimer’s Disease Neuroimaging Initiative (AD), Fisher et al. [42] found that patients had lost GM volume in both insulae, the cerebellum, the left superior temporal and parahippocampal gyri and the right thalamus and posterior cingulate gyrus prior to the onset of delusions. By comparing AD patients who did and did not develop psychoses, instead, greater GM loss over 4 years prior to onset was observed only in the right insula [43]. However, a study that focussed on delusional patients without hallucinations revealed greater longitudinal GM loss in left middle temporal and right inferior frontal and postcentral areas associated with symptom onset [44].

In summary, an integrative view of the current neuroimaging studies suggests the right frontal lobe as the primary region associated with delusional manifestations in AD, with important contributions of alterations in temporal and parietal cortices [45–47] and associated subcortical nuclei that are part of dopaminergic pathways [19, 33]. To date, however, most neuroimaging investigations have used a cross-sectional design and investigated patients with active delusions. In contrast, only a few studies have investigated the longitudinal MRI changes associated with the development of delusions. In general, these investigations were carried out on small samples of patients and neglected important potentially confounding factors such as, for example, the *ApoE* status of patients with and without delusions, since the ε4 allele is associated with increased risk of psychotic symptoms [48, 49]. Although these studies provide important insights into the neuroimaging correlates of AD-related delusions, they cannot be used to support causal inferences on the neural processes leading to the manifestation of such behavioural alterations. Their clinical utility is also limited, as signalled by the fact that recent clinical and research criteria for AD-related psychosis, although advocating for further research to clarify the underlying biological mechanisms, do not currently include neuroimaging as a potential diagnostic tool [11, 13].

Considering the heterogeneity of neuroimaging findings on the neural correlates of delusions in AD, the primary aim of this study was to ascertain the pattern of longitudinal GM degeneration associated with the manifestation of these symptoms in patients with AD. In fact, investigating brain changes leading to the onset of delusions could potentially provide mechanistic insight that could serve as a translational foundation for possible clinical therapeutic interventions by targeting selective neural pathways commonly overlooked within the current clinical context. This primary aim was pursued by comparing changes in GM volume of selected brain areas in the year prior to symptom onset in two large samples of well characterised and matched patients with and without delusions. The research hypothesis behind this study, based on the available literature, is that greater GM volume loss over the course of the year prior to symptom onset should be observed primarily in fronto-parietal cortices and/or connected GM areas in the sample of patients who did develop delusions.

The secondary aim was to investigate whether differential changes in cognition could be detected in patients with AD who did and who did not develop delusions over a year. It was hypothesised that greater cognitive decline could parallel neurodegeneration, since more severe cognitive impairment is a risk factor for AD-related psychoses [50].

## Methods

### Participants

Data used in the preparation of this article were obtained from the Alzheimer’s Disease Neuroimaging Initiative (ADNI) database (adni.loni.usc.edu).^1^ The study protocol was approved by the institutional review board of each site and all participants provided written informed consent. All data contained in the ADNI database are anonymised publicly available. Approval for secondary analyses of this dataset was granted by the Research Committee of Brunel University of London (reference number 30422-TISS-Jul/2021- 33,453-2).

**Fig. 1:**
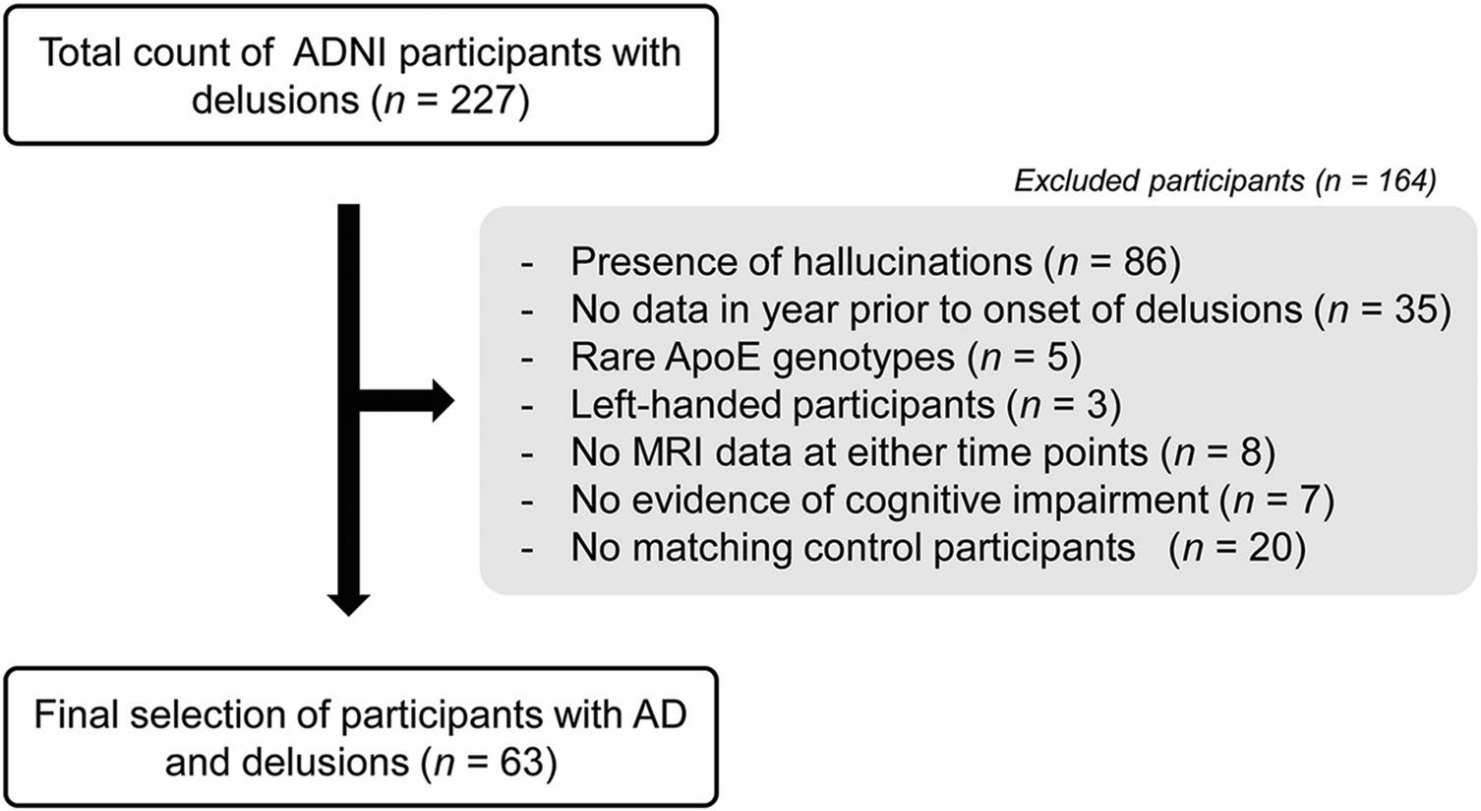
Flowchart depicting the selection process for the participants with AD and delusions included in this study

The procedure of selection of the participants included in this study is shown in Fig. 1. First, the total ADNI database was searched to identify all participants with a clinical diagnosis of either MCI or dementia and presenting with delusions (*n* = 227) recorded by means of either the Neuropsychiatric Inventory (NPI) [51] or the NPI-Questionnaire (i.e. a shorter version of the NPI) [52] at any time point. Second, the following exclusion criteria were applied to select a phenotypically characterised sample of patients with AD and delusions: presence of hallucinations (as different psychotic symptoms may be caused by partially different neural alterations [11, 15]), lack of assessments in the year before delusion onset, rare *ApoE* genotypes (i.e. ε2 carriers), left-handedness (due to possible neurostructural differences between right- and left-handed people [53]), lack of MRI data at any of the time points of interest, lack of evidence of cognitive decline (i.e. classified as cognitively unimpaired at all ADNI time points), lack of participants (either patients without delusions or healthy controls) without delusions who could be matched with the patients with delusion according to the specified matching criteria detailed below. The observation period of 1 year was selected for three reasons: (1) to detect neurocognitive changes more likely to precede and, thus, be linked to the onset of delusions, (2) to maximise sample size on the basis of data availability, (3) by considering that the annual incidence rate (over 5 years of observation) of new psychotic symptoms among patients with AD has been found to be stable at around 10% [54]. A final count of 63 people with either MCI (*n* = 29) or dementia (*n* = 34) due to AD and delusions (PT-D) were identified and matched to a sample of 63 (29 with MCI and 34 with dementia) patients with AD without delusions (PT-ND). Matching was done for diagnosis, Mini Mental State Examination (MMSE) score, age, education, sex, handedness and *ApoE* genotype. These variables were used for a one-to-one matching process that was aimed at minimising the potential confounding impact of disease severity, demographic and genetic variables that can affect clinical and neural decline. None of the patients with MCI progressed to dementia during the year of observation. Additionally, 63 healthy controls (HC) were selected and matched to the patients’ groups for age, education, sex, handedness and *ApoE* genotype.

### Clinical and cognitive data

Neuropsychiatric symptoms were assessed by means of either the NPI or the NPI-Q in different ADNI waves. Differently from the NPI-Q, the NPI assessment also includes information about the frequency of symptoms to calculate the total score. Therefore, to quantify global severity of neuropsychiatric manifestations homogenously across participants, we converted NPI scores into NPI-Q-like scores by summing up only the severity score for each symptom without multiplying it for the frequency score. Information on cardiovascular risk factors was also extracted, since previous research has highlighted a potential impact of cerebrovascular damage on risk of psychosis in AD. Scores for the following neuropsychological tests were extracted, for the PT-D group, about 1 year prior to delusion onset (Time 1) and at delusion onset (Time 2) and, for the PT-ND and HC groups, at two time points one year apart from one another: MMSE, Clock Drawing Test (CDT, drawing and copy), Trail Making Test (TMT, part A and B), Logical Memory Test (LMT, Immediate and Delayed Recall), Category Fluency Test (CFT–animals). Details of the clinical, neuropsychiatric and cognitive assessments are available at http://adni.loni.usc.edu/methods.

### AD biomarkers

Data on two biomarkers of AD pathological processes, i.e. cerebrospinal fluid (CSF) levels of β-Amyloid (Aβ) and phosphorylated tau (p-tau) [55], were also extracted at both time points for all those participants who had undergone a lumbar puncture. The relevant methods have been described in detail by previous publications [56]. Levels of CSF Aβ were considered abnormal when below a cut-off of 977 pg/mL, while values of the p-tau/Aβ ratio were categorised as pathological when above the cut-off of 0.025 [57].

### MRI data and pre-processing

Two T1-weighted MRI scans were selected for each participant: one at delusion onset and one in the year prior to onset. Details on the MRI data collection protocol have been published by Jack Jr et al. [58]. Scans were acquired at different MRI scanner field strengths, either 1.5 T or 3 T, but participants groups were matched for this variable (see Supplementary materials for details). MRI data were pre-processed and analysed using the most updated analytical pipeline of the standard voxel-based morphometry (VBM) procedure [59] implemented with Statistical Parametric Mapping 12 (Wellcome Centre for Human Neuroimaging, London, UK). In particular, the Computational Anatomy Toolbox (CAT12, http://www.neuro.uni-jena.de/cat/) was used to implement a longitudinal pre-processing pipeline optimised to detect large changes (such as neurodegeneration). The following steps were carried out (1) reorientation of images to the bi-commisural axis, (2) inverse-consistent co-registration bias-correction of the scans of each participant, (3) and segmentation, (4) modulation (by means of the Jacobian determinant of the deformation), normalisation and registration to the MNI space, and (5) smoothing with an 8 mm full-width at half maximum Gaussian kernel. Only GM maps were used to answer the research question of this study.

Finally, GM volumes were extracted from 30 regions of interest (ROIs) using the Automated Anatomical Labelling (AAL) atlas 2 [60]. These ROIs were selected on the basis of what emerged from previous studies that have investigated the neural alterations associated with delusions in AD: 6 ROIs in the dorsolateral prefrontal cortex, i.e. bilateral inferior, middle and superior frontal gyri; 4 ROIs in the medial temporal lobe, i.e. bilateral hippocampi and parahippocampal gyri; 8 ROIs in the DMN, i.e. bilateral inferior parietal lobules, precunei, posterior cingulate and medial prefrontal cortex; and 12 ROIs in the nigrostriatal pathway, i.e. substantia nigra (SN) and bilateral caudate and putamen nuclei, and in the mesocortico-limbic pathway, i.e. ventral tegmental area (VTA), bilateral accumbens, anterior cingulate and orbito-frontal cortices. The SN and VTA ROIs were defined following the methods used in previous research [61–64].

### Statistical analyses

Cognitive and clinical data were compared at baseline across all three groups to characterise the clinical profile of patients with ANOVA and FDR-corrected post hoc tests using SPSS version 26 (IBM, Chicago, IL, USA). Frequencies of participants with positive AD biomarkers (Aβ and p-tau) were compared across groups using the Chi-square test.

Repeated-measures ANCOVA models were used to investigate the primary aim, i.e. differences in longitudinal GM changes in all ROIs between the two patient groups (group × time interactions). The significance threshold was set at *p* < 0.05 with a False Discovery Rate (FDR) correction for multiple testing. Three covariates were included in the models: total intracranial volume, MRI scanner field strength and an index of longitudinal change in NPI scores. Total intracranial volume was used as a proxy measure of brain reserve [65] to rule out the possible confounding influence of this variable. MRI scanner field strength was included to account for any potential difference in acquisitions across centres, because MRI data were acquired either at 1.5 T or 3 T. It must be stressed that the three groups were matched for this parameter and that previous methodological studies using the ADNI dataset found that pooling together data acquired at different MRI scanner field strengths does not significantly affect reproducibility and reliability of findings of brain volumetric analyses [66, 67].

Finally, an index that could capture change in the total NPI score as a proportion of (i.e. corrected by) the baseline NPI score was calculated using the following procedure: (1) NPI scores at Time 1 and Time 2 were first subtracted from the maximum NPI score (i.e. 36), this step was needed, because some participants had a NPI score of 0 at Time 1 that would have made it impossible to calculate the proportion of NPI change over time; (2) a proportion of NPI change was calculated using the following formula: ((36—NPI_Time1_)—(36—NPI _Time2_))/(36—NPI _Time1_).

Post hoc VBM analyses were carried out to investigate whole-brain GM volume changes (group × time interaction effects), as well as to quantify GM atrophy in the patient groups compared to HC at baseline.

Finally, the secondary aim was investigated using repeated-measures ANCOVA models to quantify group × time interaction effects on cognitive performance in the two patient groups, using the index of NPI change as a covariate (*p* < 0.05, FDR-corrected).

## Results

At baseline, patients in the PT-D and PT-ND groups showed worse cognitive performance, higher NPI scores and lower GM fraction (i.e. GM volume divided by total intracranial volume) than HC (Table 1). The two patient groups were matched for all characteristics, but the PT-D group presented with higher NPI scores already 1 year before the manifestation of delusions. No significant differences were found in rates of cardiovascular risk factors across groups (Supplementary materials Table S2).

**Table 1:** Differences in characteristics between participant groups at Time 1 (mean and SD)

Characteristics	PT-D (*n* = 63)	PT-ND (*n* = 63)	HC (*n* = 63)	*F*	*p*
Age (years)	75.67 (6.16)	75.54 (5.90)	75.06 (5.81)	0.18	0.84
Sex (F/M)^a^	26/37	26/37	26/37	0.00^b^	1.00
*ApoE* status (ε3ε3/ε3ε4/ε4ε4^a^	19/31/13	19/33/11	19/39/5	4.60^b^	0.33
Ethnicity^c^	2/61	3/60	2/61	0.30^b^	0.86
Race^d^	3/60/0	1/61/1	4/58/1	2.83^b^	0.59
Education (years)	15.49 (2.88)	15.22 (3.28)	15.73 (2.55)	0.49	0.62
NPI	4.11 (3.63)^e^	2.30 (3.02)^e^	0.68 (1.39)	22.96	1.22 × 10^−9^
MMSE	25.10 (3.72)	25.14 (3.31)	29.32 (0.76)	43.78	2.62 × 10^−16^
CDT–drawing	3.94 (1.09)	3.90 (1.17)	4.68 (0.62)	12.42	9.00 × 10^−6^
CDT–copy	4.49 (0.93)	4.54 (0.86)	4.83 (0.42)	3.45	0.34
LMT–IR	6.02 (3.30)	6.32 (4.41)	14.52 (3.43)	104.45	3.90 × 10^−31^
LMT–DR	2.94 (3.06)	3.29 (4.09)	13.49 (3.63)	172.92	3.70 × 10^−43^
CFT–animals	13.40 (4.63)	13.46 (5.33)	20.87 (5.38)	44.29	1.85 × 10^−16^
TMT-A (sec)	52.62 (29.09)	46.10 (26.67)	35.76 (11.13)	8.12	4.15 × 10^−4^
TMT-B (sec)	174.06 (88.92)	144.84 (83.06)	84.42 (39.21)	23.85	6.10 × 10^−10^
GMF	0.38 (0.02)	0.38 (0.03)	0.40 (0.03)	12.52	0.08 × 10^−4^
WMF	0.32 (0.03)	0.32 (0.02)	0.33 (0.02)	2.70	0.07
TIV (ml)	1498.10 (172.26)	1492.14 (178.00)	1479.95 (156.17)	0.19	0.83
Aβ	31 (*n* = 37)^f^	37 (*n* = 43)^f^	16 (*n* = 32)^f^	14.99	0.001
p-tau	31 (*n* = 37)^f^	37 (*n* = 43)^f^	13 (*n* = 32)^f^	22.54	0.013 × 10^−4^

*Aβ* amyloid beta, *CDT* Clock Drawing Test, *CFT* Category Fluency Test, *GMF* grey matter fraction, *LMT–DR/IR* Logical Memory Test–delayed recall/immediate recall, *MMSE* Mini Mental State Examination, *NPI* Neuropsychiatric Inventory, *p-tau* phosphorylated tau, *TIV* total intracranial volume, *TMT–A/B* Trail Making Test–part A/part B, *WMF* white matter fraction

^a^Frequencies

^b^Chi-squared

^c^Hispanic or Latino/Not Hispanic or Latino

^d^Black or African American/White/Two or more races

^e^Significant PT-D vs PT-ND difference, *p* = 0.001 (FDR-corrected)

^f^Frequency of participants with positive biomarker status (number of participants with available biomarker data)

The time between Time 1 and Time 2 assessments was about a year for all participant groups (PT-D: 347.41 ± 77.52 days, PT-ND: 398 ± 243.40 days, HC: 397.25 ± 99.93 days) and no significant differences were observed across groups (*F* = 2.125, *p* = 0.122).

Very similar rates of participants with abnormal Aβ and p-tau levels were observed in sub-samples of the two patient groups with available biomarker data and no significant differences between patient groups were found either at Time 1 or Time 2 (Table 1 and Supplementary materials Table S3). Rates of healthy controls with positive biomarkers were significantly lower than those observed in the patient groups at both time points.

### Primary analyses—GM ROIs

**Fig. 2:**
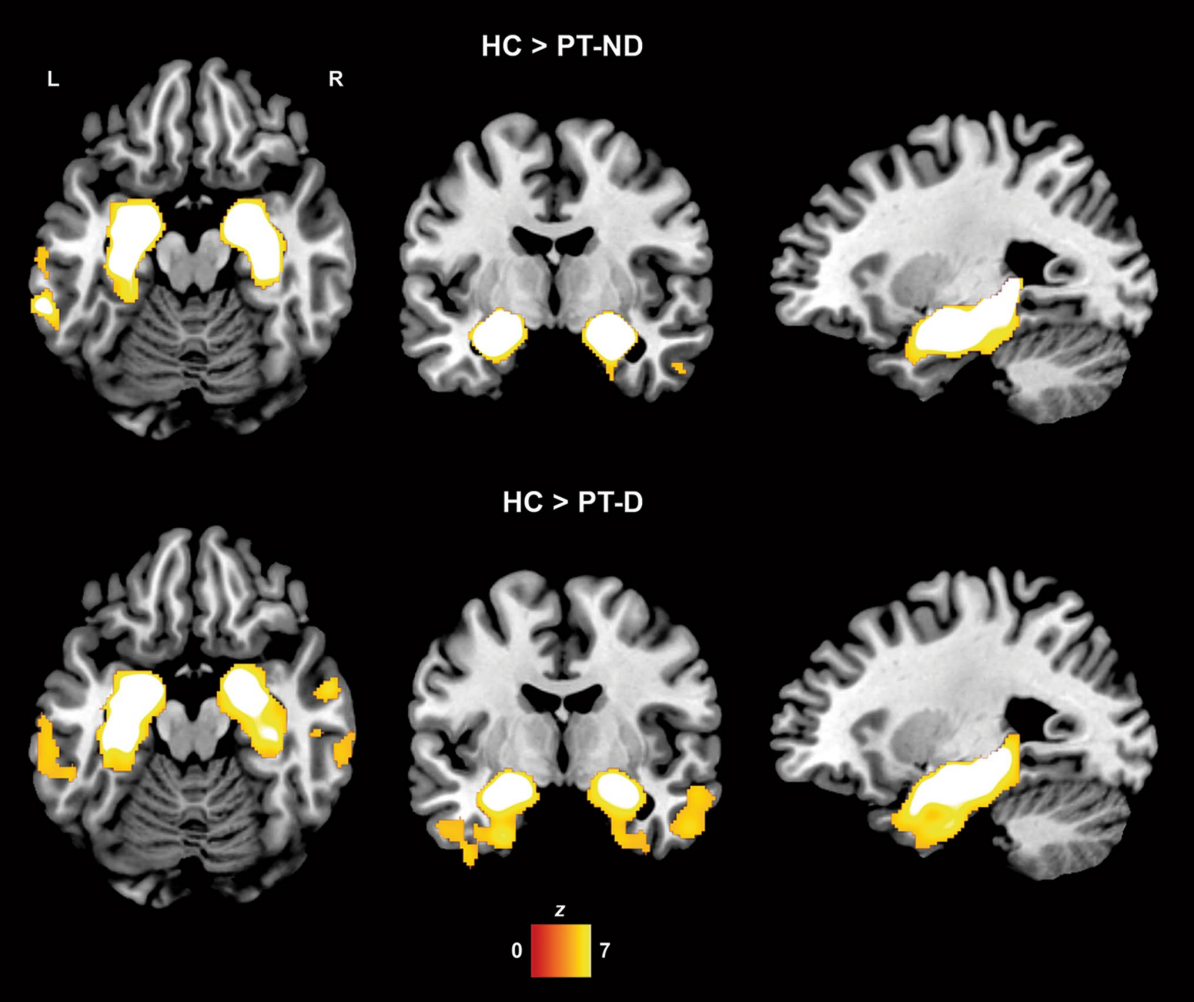
Baseline GM atrophy in patient groups compared to HC (cluster-level FWE-corrected *p* = 0.05)

Repeated-measures ANCOVA analyses of GM ROIs carried out on the patient groups highlighted significantly greater GM volume loss in both the left (*F* = 8.479, *p* = 0.004) and the right caudate nuclei (*F* = 12.204, *p* < 0.001) in the patients who subsequently developed delusions (Table 2). Similarly, greater longitudinal GM loss in delusional patients was also observed in the bilateral medio-temporal ROIs (bilateral parahippocampal gyri and left hippocampus), in the right anterior cingulate cortex and in posterior hubs of the DMN (bilateral precuneus and left posterior cingulate cortex) (Fig. 2). No significant differences in GM volume changes were observed for any of the other ROIs.

**Table 2:** Group × time interaction effects on the volumes of selected GM ROIs (mean and SD)

ROI	PT-D (*n* = 63)		PT-ND (*n* = 63)		*F*	*p*
	Time 1	Time 2	Time 1	Time 2		
Dorsolateral prefrontal cortex						
Left inferior frontal gyrus	12.15 ± 1.74	11.85 ± 1.82	12.22 ± 1.66	12.01 ± 1.65	4.245	0.041
Right inferior frontal gyrus	11.89 ± 1.64	11.61 ± 1.61	11.97 ± 1.68	11.77 ± 1.67	3.344	0.070
Left middle frontal gyrus	10.42 ± 1.69	10.17 ± 1.75	10.42 ± 1.61	10.24 ± 1.64	1.765	0.187
Right middle frontal gyrus	11.05 ± 1.68	10.81 ± 1.70	10.91 ± 1.53	10.75 ± 1.57	1.445	0.232
Left superior frontal gyrus	6.84 ± 1.11	6.72 ± 1.42	6.92 ± 0.93	6.85 ± 0.95	2.087	0.151
Right superior frontal gyrus	8.51 ± 1.39	8.31 ± 1.40	8.48 ± 1.26	8.34 ± 1.25	0.877	0.351
Medio-temporal lobe						
Left hippocampus	2.68 ± 0.45	2.58 ± 0.46	2.66 ± 0.47	2.62 ± 0.48	8.523^a^	0.004^a^
Right hippocampus	2.82 ± 0.51	2.72 ± 0.53	2.79 ± 0.48	2.72 ± 0.48	4.476	0.036
Left parahippocampal gyrus	2.77 ± 0.45	2.67 ± 0.47	2.78 ± 0.53	2.73 ± 0.53	8.867^a^	0.004^a^
Right parahippocampal gyrus	3.58 ± 0.56	3.47 ± 0.57	3.55 ± 0.53	3.50 ± 0.53	7.040^a^	0.009^a^
Default mode network						
Left posterior cingulate cortex	1.34 ± 0.26	1.30 ± 0.26	1.36 ± 0.26	1.34 ± 0.26	6.670^a^	0.011^a^
Right posterior cingulate cortex	0.64 ± 0.12	0.63 ± 0.12	0.64 ± 0.11	0.64 ± 0.11	3.367	0.059
Left precuneus	10.30 ± 1.72	10.04 ± 1.70	10.36 ± 1.77	10.28 ± 1.74	6.810^a^	0.010^a^
Right precuneus	9.18 ± 1.59	8.93 ± 1.60	9.27 ± 1.65	9.19 ± 1.63	6.618^a^	0.011^a^
Left inferior parietal lobule	5.20 ± 0.73	5.10 ± 0.78	5.26 ± 0.82	5.16 ± 0.84	0.004	0.952
Right inferior parietal lobule	3.10 ± 0.50	3.02 ± 0.53	3.15 ± 0.55	3.08 ± 0.57	0.148	0.701
Left medial prefrontal cortex	2.24 ± 0.41	2.18 ± 0.39	2.24 ± 0.30	2.20 ± 0.29	1.543	0.217
Right medial prefrontal cortex	2.02 ± 0.36	1.98 ± 0.34	2.04 ± 0.27	2.01 ± 0.26	2.010	0.159
Dopaminergic pathways						
Substantia nigra	0.02 ± 0.01	0.02 ± 0.01	0.02 ± 0.01	0.02 ± 0.01	1.125	0.291
Left caudate	2.54 ± 0.41	2.50 ± 0.42	2.49 ± 0.40	2.49 ± 0.40	8.479^a^	0.004^a^
Right caudate	2.71 ± 0.48	2.69 ± 0.51	2.69 ± 0.43	2.70 ± 0.44	12.204^a^	< 0.00^a^
Left putamen	3.58 ± 0.44	3.47 ± 0.48	3.42 ± 0.47	3.38 ± 0.50	4.891	0.029
Right putamen	3.71 ± 0.49	3.59 ± 0.53	3.61 ± 0.46	3.54 ± 0.049	2.470	0.119
Ventral tegmental area	0.01 ± 0.00	0.01 ± 0.00	0.01 ± 0.00	0.01 ± 0.00	2.199	0.141
Left nucleus accumbens	0.15 ± 0.02	0.14 ± 0.02	0.14 ± 0.02	0.14 ± 0.02	3.949	0.049
Right nucleus accumbens	0.25 ± 0.03	0.24 ± 0.04	0.25 ± 0.04	0.25 ± 0.04	5.516	0.020
Left anterior cingulate cortex	4.18 ± 0.62	4.05 ± 0.62	4.11 ± 0.53	4.03 ± 0.55	5.820	0.017
Right anterior cingulate cortex	3.66 ± 0.55	3.55 ± 0.54	3.62 ± 0.49	3.55 ± 0.51	6.901^a^	0.010^a^
Left orbitofrontal cortex	2.51 ± 0.46	2.45 ± 0.45	2.48 ± 0.35	2.44 ± 0.35	3.959	0.049
Right orbitofrontal cortex	2.35 ± 0.43	2.28 ± 0.42	2.33 ± 0.32	2.28 ± 0.32	4.863	0.029

^a^Significant interaction effect surviving FDR correction for multiple testing

**Fig. 3:**
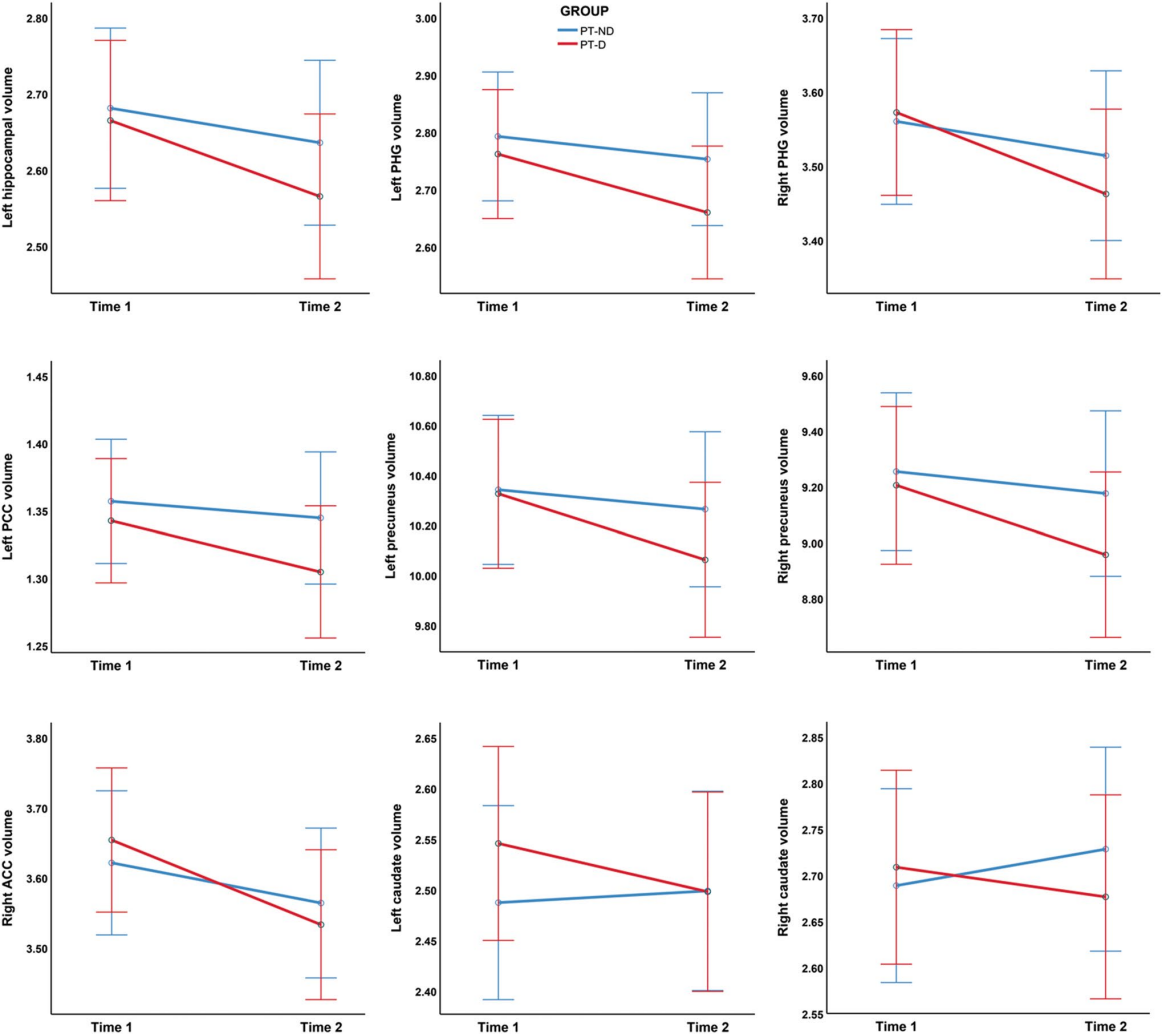
Regions of interest showing significantly greater GM loss (group × time interaction effect) in PT-D compared with PT-ND

Whole-brain repeated-measures models revealed no significant differences in GM degeneration over time in the PT-D vs PT-ND comparison. At baseline, no regional GM volume differences were observed between patient groups who both showed similar patterns of medio-temporal GM atrophy in comparison to HC (Fig. 3).

### Secondary analyses—cognitive performance

Longitudinal decline in cognitive performance was greater in both patient groups when compared with HC across functions, but significantly greater decline was observed only for verbal delayed recall (*F* = 5.136, *p* = 0.025) in the PT-D compared with the PT-ND group (Supplementary materials Table S4).

## Discussion

In this study, patients with AD who developed delusions over a year showed greater GM loss than patients who did not develop delusions in subcortical and cortical regions that are part of dopaminergic pathways, as well as in medial temporo-parietal areas. These longitudinal changes were mainly seen in the right caudate nucleus, a target of the nigrostriatal dopaminergic pathway, despite no detectable volumetric differences between PT-D and PT-ND 1 year prior to delusion onset. The two samples were carefully selected and represent, to the best of our knowledge, some of the largest ever to be used to investigate this research question including patients matched for potentially confounding demographic and genetic characteristics.

These findings suggest that delusions may result from a prominent involvement of widespread subtle structural damage in the brain regions that are part of dopaminergic pathways and in areas of the medio-temporal lobe and of the DMN, that are typically affected by AD pathology. In particular, accelerated GM loss in the right caudate nucleus was especially evident in the PT-D group. Previous accounts have been published implicating caudate damage in delusional thoughts both in people with [68] and without AD: lacunar stroke in the right caudate associated with frontal hypometabolism [69] and left caudate infarction in the absence of dementia [70, 71]. Moreover, lower caudate volume was observed in early-stage unmedicated patients with schizophrenia [72], thus suggesting a role for dysfunction in the dorsal striatum in relation to the emergence of psychotic symptoms. Lower dorsal striatum volume has been previously found associated with lower dopamine receptor availability [73, 74] and appears to be linked to higher likelihood of odd beliefs in people with no psychiatric conditions, although volumetric alterations were mainly detected in the putamen [75]. Moreover, greater GM loss was detected in the PT-D group also in the right anterior cingulate, a cortical target of the mesocortical dopaminergic pathway, in line with previous accounts [27, 33, 39]. This finding suggests that a degree of right-lateralised frontal damage may indeed contribute to delusion onset in patients with AD. In fact, this brain region is thought to be involved in several executive cognitive functions, including performance-monitoring and regulation of attention [76], that may contribute to belief formation. Activation in both the anterior cingulate and the caudate nucleus has been found to be associated with value attribution during multiple choice tasks [77]. Connectivity between the caudate nucleus and several prefrontal areas is also crucial to support episodic and working memory performance that appears to be mediated by dopamine levels following an inverted-U-shaped function [78, 79]. Hence, alterations in this fronto-striatal system caused by AD-related neurodegeneration may drive aberrant value/meaning attribution leading to the formation and acceptance of false beliefs [80].

However, whether dopaminergic dysfunction contributed substantially to the GM loss observed in the caudate nuclei and the right anterior cingulate cortex of the PT-D group cannot be determined within the context of this study. In fact, although previous research found that normal belief formation in healthy participants [81] is associated with dopaminergic function, delusions in people with schizophrenia [72, 82] and AD [37] appear to be mainly caused by a striatal hyperdopaminergic state. A potential reconciliation between our findings and previous evidence about the relationship between dopaminergic upregulation and psychotic symptoms may come from the observation that elevated levels of dopamine have been found to be neurotoxic and cause neuronal death under certain conditions [83]. Therefore, a condition of striatal hyperdopaminergia, potentially caused by severe AD-related cholinergic alterations in the PT-D group [84], might have driven an acceleration in GM loss. Additionally, we could not rule out the potential impact of Lewy body pathology as the driving factor of dopaminergic alterations and, in turn, neurodegeneration [85]. Lewy body pathology, in fact, has been found to be associated with higher risk of delusions in people with AD [16]. Hence, the accumulation of Lewy body pathology in the dopaminergic pathways might represent one of the factors contributing to greater subcortical GM loss and predisposing some patients with AD to manifest delusional thoughts. These interpretational avenues are speculative, however, and cannot be tested with the data available in the ADNI dataset, but will require future prospective testing.

The PT-D group presented also with accelerated GM loss in areas of the medio-temporal lobe bilaterally and in the posterior DMN, consistently with findings from previous similar studies [16, 22, 39, 44]. It is worth noting that medio-temporal regions are connected to the striatum [86] and damage to these systems, as highlighted by our analyses, could be involved in delusional beliefs, especially of the misidentification type, due to the role of medio-temporal structures in memory recollection (hippocampus) and context attribution (parahippocampal gyrus) [26]. The medio-temporal lobe is also tightly connected with the DMN [87], a functional brain network that has already been found particularly altered in patients with AD and delusions [25]. On the basis of the findings of this study, it may be hypothesised that a combination of damage in the cingulo-striatal and in the medio-temporal DMN systems may represent a more comprehensive mechanistic explanation of the genesis of delusions in AD [15].

In contrast, no structural alterations were observed in the dorsolateral prefrontal cortex in the PT-D group. It is possible that structural alterations in this part of the frontal lobe may manifest at a later stage and be linked to persistence of symptoms, given their involvement in reality monitoring processes necessary to discard wrong beliefs on the basis of new evidence [88]. Alternatively, the emergence of delusions in AD may be influenced by functional alterations, rather than GM loss, in dorsolateral prefrontal areas that were not investigated as part of this study.

Moreover, the PT-D group also showed greater decline than the PT-ND group in episodic memory (i.e. the delayed recall of the LMT) and in their behavioural profile (i.e. NPI score). These clinical findings appear to be consistent with an interpretation of a potential acceleration in neurodegeneration and, as a consequence, of symptoms that may have contributed to the emergence of delusions.

This study has several strengths: it represents one of the largest studies to date assessing the structural neuroimaging correlates of delusions in AD; it provides a longitudinal investigation of GM changes associated with the emergence of this symptom by directly comparing two samples of participants with AD; participants in all groups were carefully matched to control for all main potential confounders, including *ApoE* genotype. However, some limitations must also be taken into considerations: (1) the presence of different delusion subtypes, potentially associated with partially different neural correlates and with different prognoses [89], could not be investigated due to lack of detailed phenomenological description of symptoms in the ADNI cohort; (2) delusions had been assessed by means of the NPI/NPI-Q completed by participants’ partners, a mode of assessment that may lead to some misdiagnosis of delusions as a consequence of agitation, confusion and AD-related cognitive decline [11, 90]; (3) the lack of neuroimaging data that could provide useful insights on dopamine pathways (e.g. positron emission tomography) limits our interpretation of the causal association between dopaminergic dysfunction and neurodegeneration and delusion onset, since different dopaminergic neurons and receptors may be differentially involved in psychotic symptoms and even interact with other neurotransmitters; (4) differences in severity of AD pathology between the two patient groups cannot be fully ruled out given the lack of AD biomarker data for some participants; however, no significant differences in positivity rates for either Aβ or p-tau were found between the two sub-groups of patients for whom CSF biomarkers were available.

This study detected greater neurodegeneration in cingulo-striatal and medial temporo-parietal regions in people with AD and delusions than in those without, thus suggesting that a complex and multifaceted neuropathological process may be involved in the development of delusions. Dopaminergic dysfunction might contribute to GM loss in the caudate nuclei and in the anterior cingulate and interact with damage in medio-temporal and posterior DMN areas. However, future investigations are needed to understand the relationship between GM loss within and outside dopaminergic pathways and functional brain alterations (e.g. in metabolism, perfusion, resting-state functional connectivity and brain activation) that may represent earlier and more sensitive predictors of delusions in AD. Identifying possible differences in neuroimaging markers of subtypes of delusions may help clarifying the cause of worse prognosis observed in patients with AD and misidentification delusions [89]. Moreover, the effects of cognitive and brain reserve on the emergence of neuropsychiatric symptoms may offer insights on inter-individual variability in cognitive and behavioural manifestations [65]. In addition, these findings, if confirmed in future investigations, may contribute to provide evidence supporting the use of antipsychotic medications acting on the dopaminergic system, like some of the currently available antipsychotics (e.g. aripiprazole and risperidone). However, these treatments are accompanied by important side effects and new compounds must be designed with improved safety and effectiveness profiles [15]. Finally, but crucially, considerable developments are needed in the conceptualisation and modelling of the cognitive and neural computations involved in belief formation [91, 92], as well as their alterations [80], to advance our understanding of the complex mechanisms that underpin delusions across neurological and psychiatric conditions.

## Supplementary Information

Below is the link to the electronic supplementary material.

Supplementary file1 (DOCX 21 KB)

## Data Availability

All ADNI data are publicly available at adni.loni.usc.edu.

## Footnotes

1 The ADNI was launched in 2003 as a public–private partnership, led by Principal Investigator Michael W. Weiner, MD. The primary goal of ADNI has been to test whether serial magnetic resonance imaging (MRI), positron emission tomography (PET), other biological markers, and clinical and neuropsychological assessment can be combined to measure the progression of mild cognitive impairment (MCI) and early Alzheimer’s disease (AD). For up-to-date information, see www.adni-info.org.
