# Supplementary material for: Accelerated atrophy in dopaminergic targets and medial temporo-parietal regions precedes the onset of delusions in patients with Alzheimer’s disease
Source: Eur Arch Psychiatry Clin Neurosci. 2022 May 13;273(1):229–41. doi: 10.1007/s00406-022-01417-5 (PMC9958148; doi:10.1007/s00406-022-01417-5)
Supplement: Supplementary file 1 — Supplementary file1 (DOCX 21 KB) [file 406_2022_1417_MOESM1_ESM.docx]

**Table S1.** Frequencies of T1-weighted scan acquired at different MRI scanner field strength at both time points.

| **MRI scanner field strength** | **PT-D (*n* = 63)** | **PT-ND (*n* = 63)** | **HC (*n* = 63)** | **χ^2^** | ***p*** |
| --- | --- | --- | --- | --- | --- |
| *Time 1* |  |  |  |  |  |
| 1.5T/3T | 41/22 | 41/22 | 43/20 | 0.189 | 0.910 |
| *Time 2* |  |  |  |  |  |
| 1.5T/3T | 41/22 | 37/26 | 37/26 | 0.711 | 0.701 |

**Table S2.** Rates of cardiovascular risk factors across groups.

| **Cardiovascular risk factors** | **HC (n = 61)** | **PT-ND (n = 62)** | **PT-D (n = 63)** | **χ^2^** | **p** |
| --- | --- | --- | --- | --- | --- |
| Any cardiovascula risk factor | 44 (72.1%) | 39 (62.9%) | 48 (76.2%) | 2.78 | 0.25 |
| Hypertension | 28 (45.9%) | 27 (43.5%) | 41 (65.1%) | 6.99 | 0.03 |
| Carotid artery disease | 4 (6.6%) | 6 (9.7%) | 6 (9.5%) | 0.48 | 0.78 |
| Arterial stenosis | 1 (1.6%) | 3 (4.8%) | 1 (1.6%) | 1.64 | 0.44 |
| Hyperlipidaemia | 16 (26.2%) | 19 (30.6%) | 14 (22.2%) | 1.14 | 0.56 |
| Heart murmur | 1 (1.6%) | 1 (1.6%) | 2 (3.2%) | 0.47 | 0.79 |
| Myocardial infarction | 2 (3.2%) | 3 (4.8%) | 2 (3.2%) | 0.30 | 0.86 |
| Myocarditis | 0 (0.0%) | 0 (0.0%) | 1 (1.6%) | 1.96 | 0.37 |
| Valve issues | 4 (6.6%) | 1 (1.6%) | 1 (1.6%) | 3.23 | 0.20 |
| Angina | 2 (3.2%) | 1 (1.6%) | 3 (4.8%) | 0.99 | 0.61 |
| Atrial fibrillation | 7 (11.5%) | 2 (3.2%) | 3 (4.8%) | 3.92 | 0.14 |
| Heart rhythm issues | 3 (4.9%) | 3 (4.8%) | 2 (3.2%) | 0.29 | 0.86 |
| Congestive heart failure | 1 (1.6%) | 0 (0.0%) | 0 (0.0%) | 2.06 | 0.36 |
| Rheumatic heart disease | 0 (0.0%) | 0 (0.0%) | 1 (1.6%) | 1.96 | 0.37 |

**Table S3.** Between-group differences in cerebrospinal fluid AD biomarker positivity at T2.

| **Biomarkers at Time 2** | **PT-D (*n* = 63)** | **PT-ND (*n* = 63)** | **HC (*n* = 63)** | **χ^2^** | ***p*** |
| --- | --- | --- | --- | --- | --- |
| Amyloid beta | 19 (*n* = 22)^a^ | 16 (*n* = 19)^a^ | 8 (*n* = 17)^a^ | 9.222 | 0.010 |
| Phosphorylated tau | 18 (*n* = 22)^a^ | 16 (*n* = 19)^a^ | 7 (*n* = 17)^a^ | 10.138 | 0.006 |

^a^ Frequency of participants with positive biomarker status (number of participants with available biomarker data)

**Table S4.** Group × time interaction effects on cognitive performance (mean and SD).

|  | **PT-D (*n* = 63)** | | | **PT-ND (*n* = 63)** | | |  |  |
| --- | --- | --- | --- | --- | --- | --- | --- | --- |
| **Test** | **Time 1** | **Time 2** | **Time 1** | | **Time 2** | **F** | | ***p*** |
| NPI | 4.11 (3.63) | 6.35 (4.48) | 2.30 (3.02) | | 2.33 (2.54) | 16.18 | | 0.01 × 10^-2^ |
| MMSE | 25.10 (3.72) | 22.95 (4.69) | 25.14 (3.31) | | 23.71 (4.22) | 1.07 | | 0.303 |
| CDT – drawing | 3.94 (1.09) | 3.75 (1.18) | 3.90 (1.17) | | 3.68 (1.23) | 0.02 | | 0.888 |
| CDT – copy | 4.49 (0.93) | 4.30 (1.10) | 4.54 (0.86) | | 4.46 (1.06) | 0.38 | | 0.538 |
| LMT – IR | 6.02 (3.30) | 4.88 (3.60) | 6.32 (4.41) | | 5.83 (4.45) | 1.31 | | 0.254 |
| LMT – DR | 2.94 (3.06) | 1.81 (2.97) | 3.29 (4.09) | | 3.33 (4.65) | 5.14 | | 0.025 |
| CFT – animals | 13.40 (4.63) | 12.51 (5.20) | 13.46 (5.33) | | 12.95 (5.44) | 0.62 | | 0.433 |
| TMT-A (sec) | 52.62 (29.09) | 56.98 (25.66) | 46.10 (26.67) | | 54.38 (33.74) | 0.04 | | 0.834 |
| TMT-B (sec) | 174.06 (88.92) | 193.13 (89.30) | 144.84 (83.06) | | 159.49 (92.64) | 0.43 | | 0.511 |

CDT: Clock Drawing Test, CFT: Category Fluency Test, LMT – DR/IR: Logical Memory Test – Delayed Recall/Immediate Recall, MMSE: Mini Mental State Examination, NPI: Neuropsychiatric Inventory, TMT – A/B: Trail Making Test – part A/part B
